# Supplementary material for: A New Fluorescent Probe for Hydrogen Sulfide Detection in Solution and Living Cells
Source: Molecules. 2023 Aug 23;28(17):6195. doi: 10.3390/molecules28176195 (PMC10488361; doi:10.3390/molecules28176195)
Supplement: Supplementary file 1 [file molecules-28-06195-s001.zip › molecules-2474128-supplementary.pdf]

Supporting Information

Communication

A New Fluorescent Probe for Hydrogen Sulfide Detection in Solution and Living Cells

Wei Feng [1,2,*,†], Qicai Xiao [3,†], Lu Wang [4] and Yuanyong Yang [1,*]

[1] School of Pharmacy, Guizhou Medical University, Guiyang 550025, China
[2] BGI-Shenzhen, Shenzhen 518038, China
[3] School of Pharmaceutical Sciences (Shenzhen), Sun Yat-sen University; Shenzhen 510006, China; xiaoqicai@mail.sysu.edu.cn
[4] Department of Physiology, College of Basic Medicine, Guizhou Medical University; Guiyang 550025, China; wanglu@gmc.edu.cn
* Correspondence: fengweinku@sina.com (W.F.); yangyuanyong@gmc.edu.cn (Y.Y.)
† These authors contributed equally to this work.

Table of contents

1. General Information--S3
2. General procedures ---S4
3. Figures --S6
4. Spectra --S9

General information

All chemicals were obtained from commercial sources and were used as received unless otherwise noted. All the anhydrous solvents required were purchased from Energy Chemical. Reactions were monitored using precoated Aluminum supported silica gel 60 F254 TLC (thin layer chromatography) plates (Merck) and are visualized by UV light at 254 nm. The final product was purified using column chromatography (100-200 mesh silica gel purchased from Merck) and preparative HPLC of QBH. 1H NMR (600 MHz) and 13C NMR (151 MHz) spectra were recorded on the Bruker AVANCE 600 MHz spectrometer. Deuterated chloroform, methanol d4 were used as solvents, and Chemical shifts (δ) for 1Hand 13C-NMR spectra are given in ppm relative to tetramethylsilane (TMS) [δ 7.26 for 1H (chloroform-d), δ 77.0 for 13C (chloroform-d); δ 3.31 for 1H (Methanlo-d4), δ 49 for 13C (methanol-d4)], Abbreviations used in the NMR follow-up experiments: br, broad; s, singlet; d, doublet; t, triplet; q, quartet; sep, septet; dd, doublet of doublet; m, multiplet. High resolution mass spectra (HRMS) was obtained from Orbitrap Elite HybridIon Trap-Orbitrap (Thermofischer scientific, Newington, NH, USA) Mass Spectrometer in electrospray ionization mode (ESI+). The cancer cell line MCF-7 was ATCC HTB-22, and was purchased from Biobw Pte Ltd. Product link is as below: https://www.biobw.org/Cells/bio-69083.html.

General procedures:

Procedure for probe response to sodium sulfide test: The probe FL-N_3 was dissolved in methanol at a concentration of 2 mM. Sodium sulfide was dissolved in water at a concentration of 40 mM. Two wells of a corning 96-well plate were selected and to each well was added 100 μL probe solution. 100 μL Na_2S solution was added to one of the wells and to the other was added 100 μL water. The plate was incubated at RT for 20 minutes before reading fluorescence on a plate reader. The fluorescence was read at λ_{ex}=455 nm with λ_{em} set from 500 nm to 600 nm.

Procedure for fluorescence response over time test: The probe FL-N_3 was dissolved in methanol at a concentration of 10 mM. Sodium sulfide was dissolved in water at a concentration of 20 mM. To a corning 96-well plate was added 100 μL probe solution and 100 μL Na_2S solution. The fluorescence was read on a plate reader every minute in 2 hours with λ_{ex}=455 nm and λ_{em}= 515 nm.

Procedure for probe selectivity test: The probe FL-N_3 was dissolved in methanol at a concentration of 1 mM. Other tested compounds or salts were dissolved in water at a concentration of 10 mM. To a corning 96-well plate, was added 160 μL water and 20 μL probe solution. Then 20 μL aqueous solution of one tested compound or salt was added to one well. To the well of blank, 20 μL water was added. After 30-minute incubation at RT, fluorescence was read on a plate reader (brand) with λ_{ex}=455 nm and λ_{em}= 515 nm.

Procedure for linear correlation test: The probe FL-N_3 was dissolved in methanol at a concentration of 10 mM. To 6 wells of a corning 96-well plate was added 20 μL of the probe solution. Following this, to the 6 wells was added 180 μL, 160 μL, 140 μL, 100 μL, 60 μL, 20 μL water in order. Then 0 μL, 20 μL, 40 μL, 80 μL, 120 μL, 160 μL of sodium sulfide solution (1 mM) was added to the 6 wells in order. After incubation at RT for 20 minutes, the fluorescence was read on plate reader (brand) with λ_{ex}=455 nm and λ_{em}= 515 nm.

Procedure for Detection Limit test: The fluorescence emission spectrum of Probe was measured three times without Na_2S and the standard deviation was calculated. To obtain the slop, the fluorescence intensity at 515 nm was plotted against Na_2S concentrations. Detection Limit (DL) was calculated according to the equation of DL= $3\sigma/k$, σ is the standard deviation and k is the slop.

Procedure for confocal imaging: MCF7 cells were seeded at 20000 cells/well in an 8-chamber plate (0.8 cm2). Probe FL-N_3 (20 μM in DMSO) was added and cells were incubated for 2 h at 37 °C. Cells were then washed with PBS (3×), and Na_2S solution (0.1 mM) was added and incubated for 5 h. Fluorescent images of the live cells were obtained using a confocal fluorescence microscope FV 1000 and processed using Olympus Fluoview Ver.3.1. Viewer (Olympus, Tokyo, Japan).

Procedure for the synthesis of compound B: Fluorescein sodium salt (135 mg, 0.36mmol) was added to a two necked rbf, after evacuation, the rbf was recharged with N_2, anhydrous DMF (3 mL) was added followed by addition of 2,5,8,11,14,17-hexaoxanonadecan-19-yl 4-methylbenzenesulfonate (450mg, 1mmol). The mixture was heated to 90 degree and stirred for 36 hours. The mixture was cooled to room temperature, and DMF was removed by evaporation and the residual was redissolved in 10 mL 10% $NaHCO_3$ solution. Extracted with EA for 3 times and the organic layer was combined, washed with brine, dried over Na_2SO_4 and concentrated. The residual was redissolved in 5 mL methanol, 2 M NaOH (2 mL) was added, and stirred at room temperature for 2 hours. 1M HCl was added slowly to adjust PH to 2, and the mixture was stirred for 30 minutes. Methanol was removed via rotary evaporation and the residual was extracted with EA for 3 times. The combined organic layer was washed with brine, dried over Na_2SO_4 and concentrated. The product was isolated via silica gel chromatography (DCM/MeOH = 100/1) with a yield of 65%.

Procedure for the synthesis of compound C: Compound B (122mg, 0.2mmol) was dissolved in 10 mL anhydrous MeCN under Ar. Silver oxide (92 mg, 0.4 mmol) was added followed by addition of chloromethyl methyl thioether (167 μL, 2 mmol) and one drop of pyridine. The mixture was heated to 50 degree and stirred for 48 hours. Cooled to room temperature and solvent was removed via rotary evaporation. The product was isolated via silica gel chromatography (DCM/MeOH = 100/1) with a yield of 81%.

Procedure for the synthesis of probe FL-N_3: Compound C (67mg, 0.1 mmol) was dissolved in 5 mL anhydrous DCM under Ar. N-chlorosuccinimide (16 mg, 0.12 mmol) was added and the mixture was stirred at room temperature for 3 hours. Then trimethyl silane chloride (13 mg, 0.12 mmol) was added and the mixture was stirred at room temperature further for 6 hours. After removal of solvent via rotary evaporation, the residual was dried under vacuum and redissolved in dry THF under Ar, following this, $TMSN_3$ (0.2 mmol) and TBAF (0.2 mmol, 1M in THF) were added and the mixture was stirred at room temperature overnight. Remove solvent via rotary evaporation and the product was isolated via silica gel chromatography (DCM/MeOH = 100/1) as a yellow oil. The product was further purified via prep HPLC in the case of impure.

Figures:

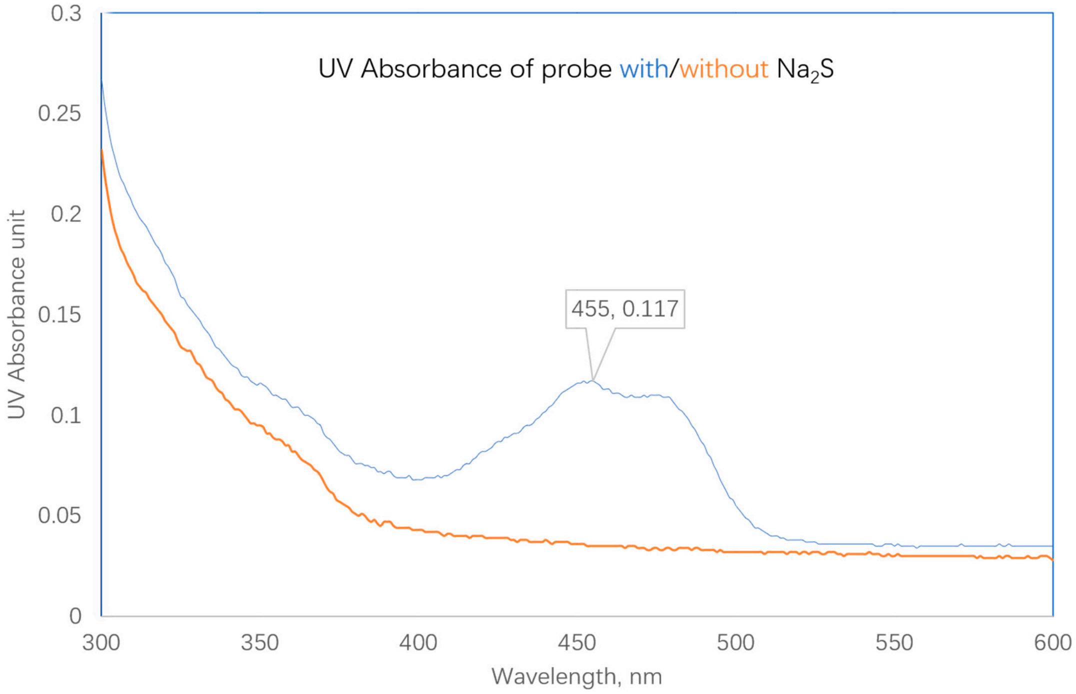

Figure S1. UV spectra analysis of the reaction mixture of FL-N_3 with Na_2S. 100 μM probe was mixed with 1 mM Na_2S in H_2O/MeOH (9/1, v/v) and the mixture was incubated at 25 °C for 20 minutes.

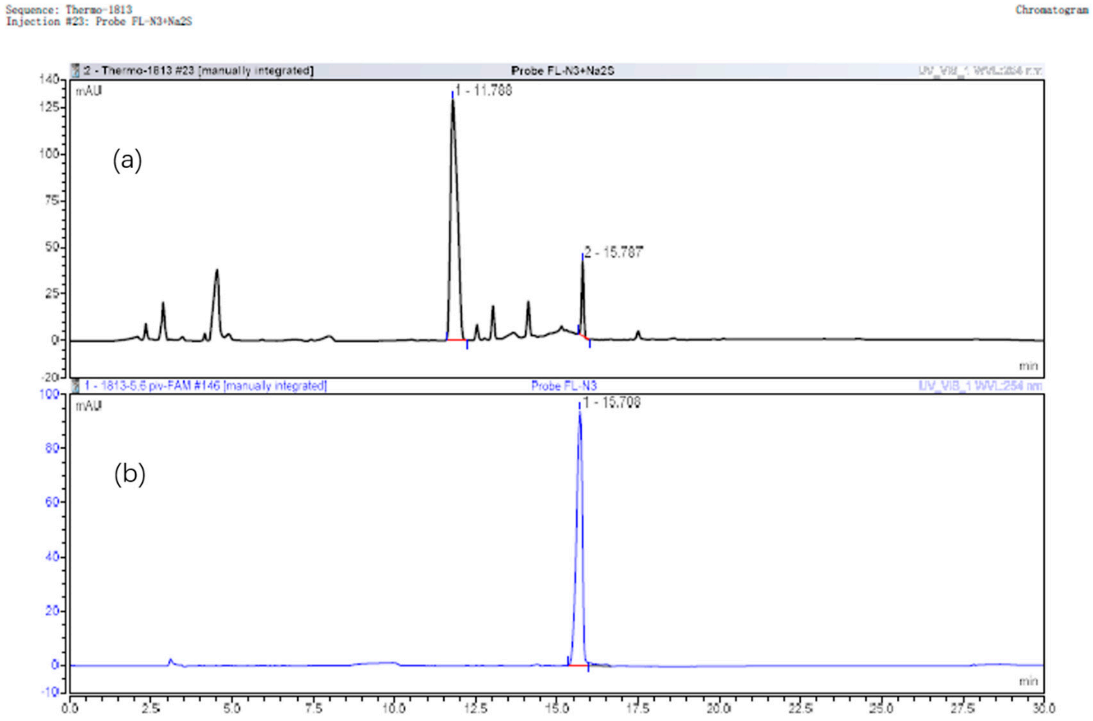

Figure S2. HPLC analysis of the reaction between FL-N_3 and Na_2S, (a) Reaction mixture of FL-N_3 (100 μM) with Na_2S (1 mM) in H_2O/MeOH (9/1, v/v) after mixed for 30 minutes; (b) Probe FL-N_3 in H_2O/MeOH (9/1, v/v).

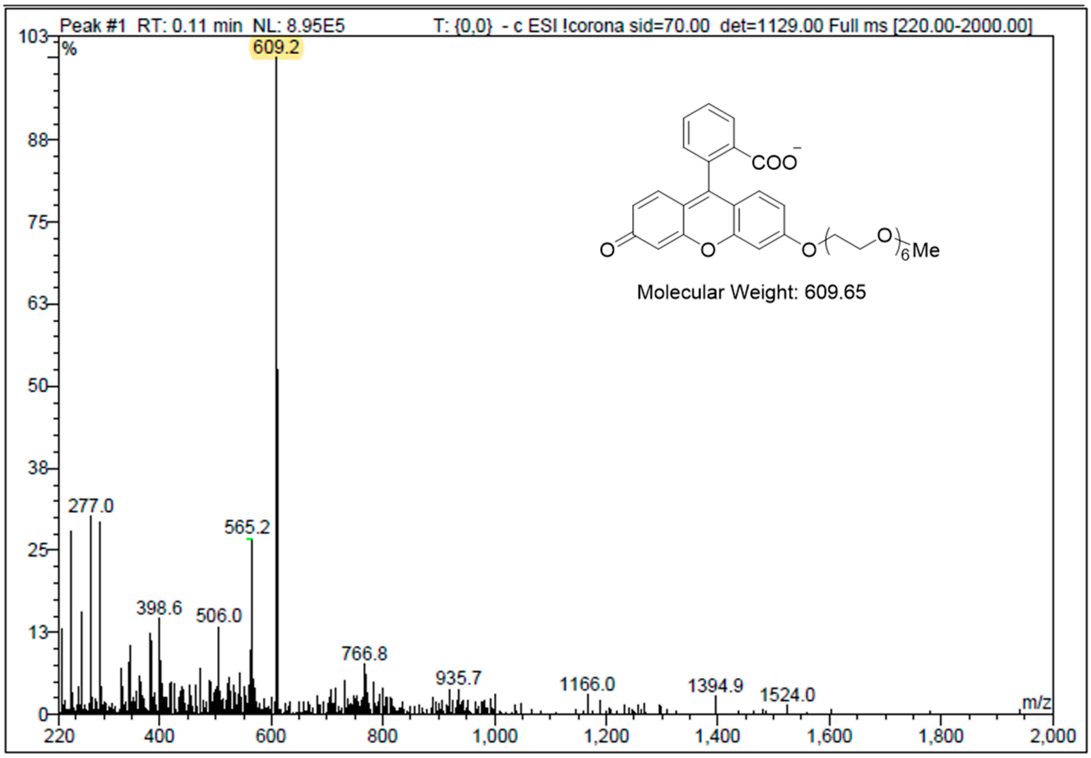

Figure S3. Mass analysis of the reaction mixture of FL-N_3 with Na_2S. Reaction mixture of FL-N_3 (100 μM) with Na_2S (1 mM) in H_2O/MeOH (9/1, v/v) was analyzed by ESI mass spectra after incubated for 30 minutes. The major product was proved to be FL-O^-.

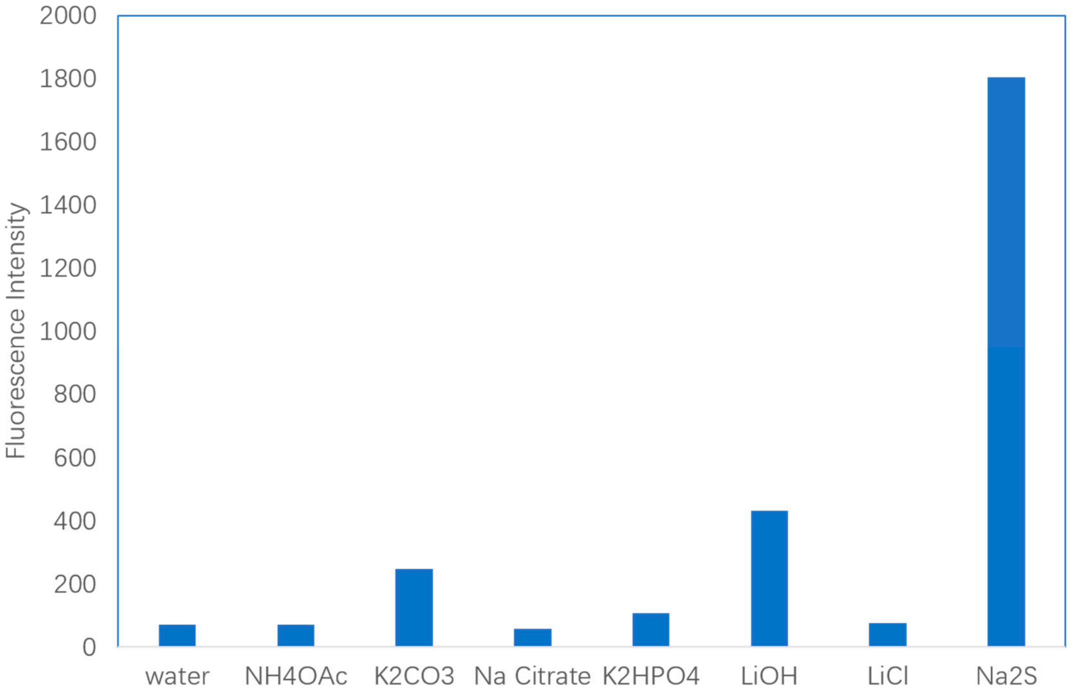

Figure S4. Fluorescence intensity of the probe (0.05 mM probe, 30 minutes after mixing) in

the presence of sulfide or other species in water and methanol (10% MeOH, v/v). 1. water, 2. Ammonium acetate, 3. Potassium carbonate, 4. Sodium citrate, 5. Potassium hydrogen phosphate, 6. Lithium hydroxide, 7 Lithium chloride, 8. **Sodium sulfide**, (2-8: 0.5 mM).

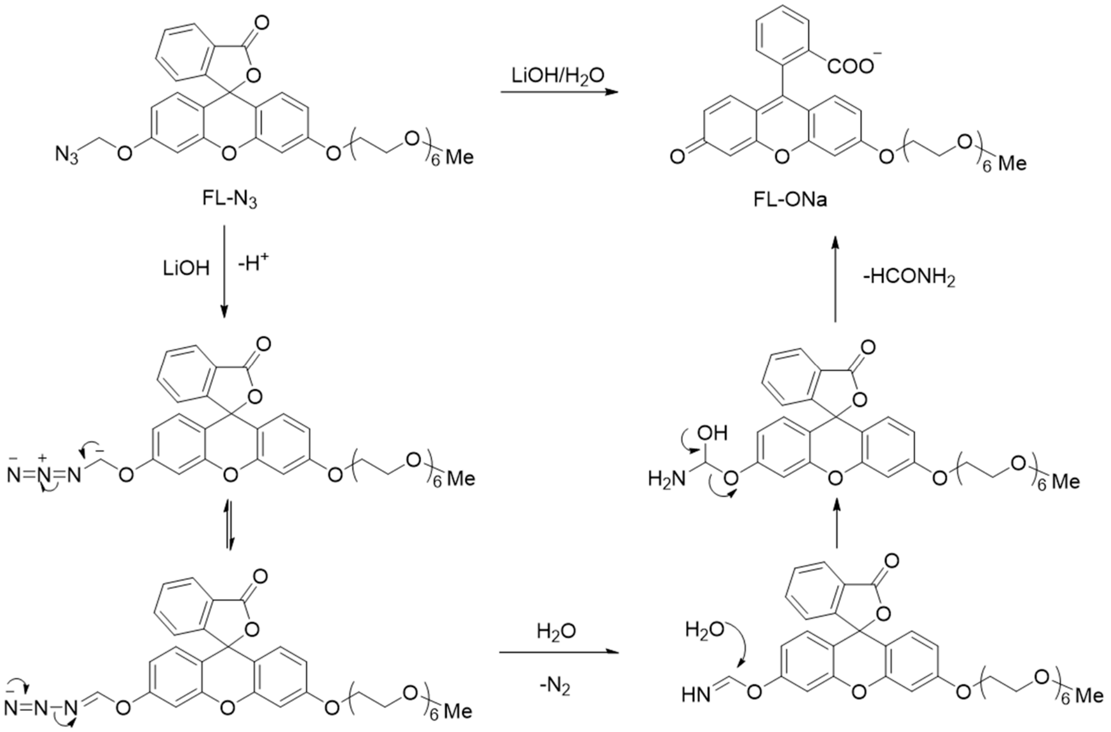

Figure S5. Proposed mechanism for base-induced fluorescence turn on.

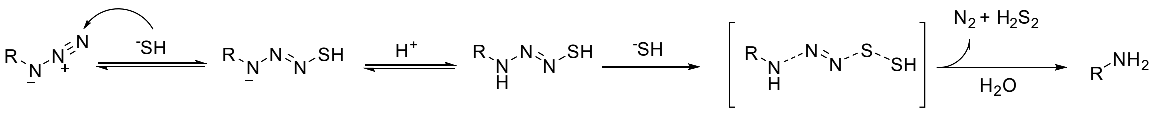

Figure S6. Possible mechanism for sulfide induced azide reduction.

Spectra:

Compound E 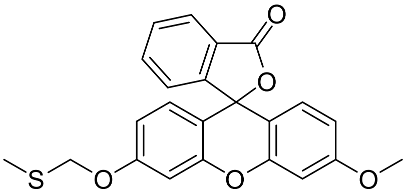

^{1}H NMR (600 MHz, $CDCl_3$) δ 8.24 (d, J = 7.8 Hz, 1H), 7.73 (td, J = 7.5, 1.1 Hz, 1H), 7.67 (td, J = 7.8, 1.0 Hz, 1H), 7.30 (d, J = 7.4 Hz, 1H), 7.01 (d, J = 2.4 Hz, 1H), 6.90 (d, J = 8.9 Hz, 1H), 6.84 (d, J = 9.7 Hz, 1H), 6.77 (dd, J = 8.9, 2.4 Hz, 1H), 6.53 (dd, J = 9.7, 1.8 Hz, 1H), 6.45 (d, J = 1.8 Hz, 1H), 5.23 (s, 2H), 3.64 (s, 3H), 2.28 (s, 3H). ^{13}C NMR (151 MHz, $CDCl_3$) δ 185.75, 165.57, 161.28, 158.85, 153.83, 149.83, 134.56, 132.68, 131.12, 130.56, 130.29, 130.18, 130.06, 129.66, 128.84, 117.96, 115.47, 114.03, 105.87, 102.50, 72.78, 52.42, 14.74. HRMS (ESI) M/Z 407.0939 ([M + H$^+$]), calculated for [$C_{23}H_{19}O_5S$ + H$^+$] 407.0953.

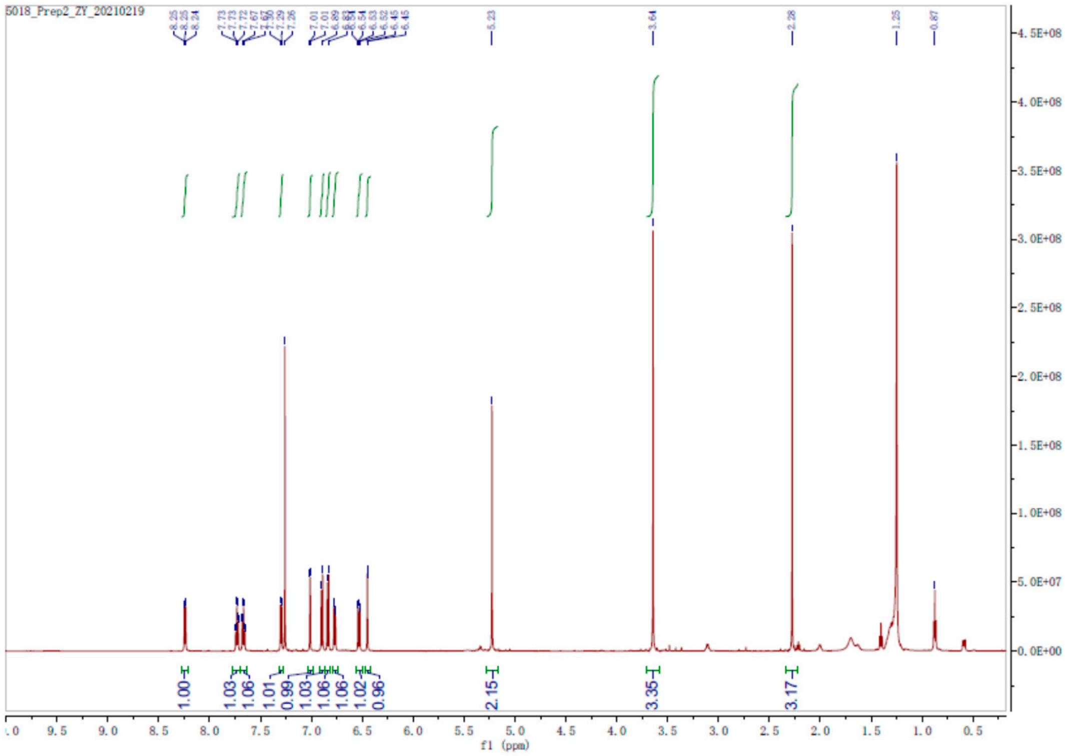

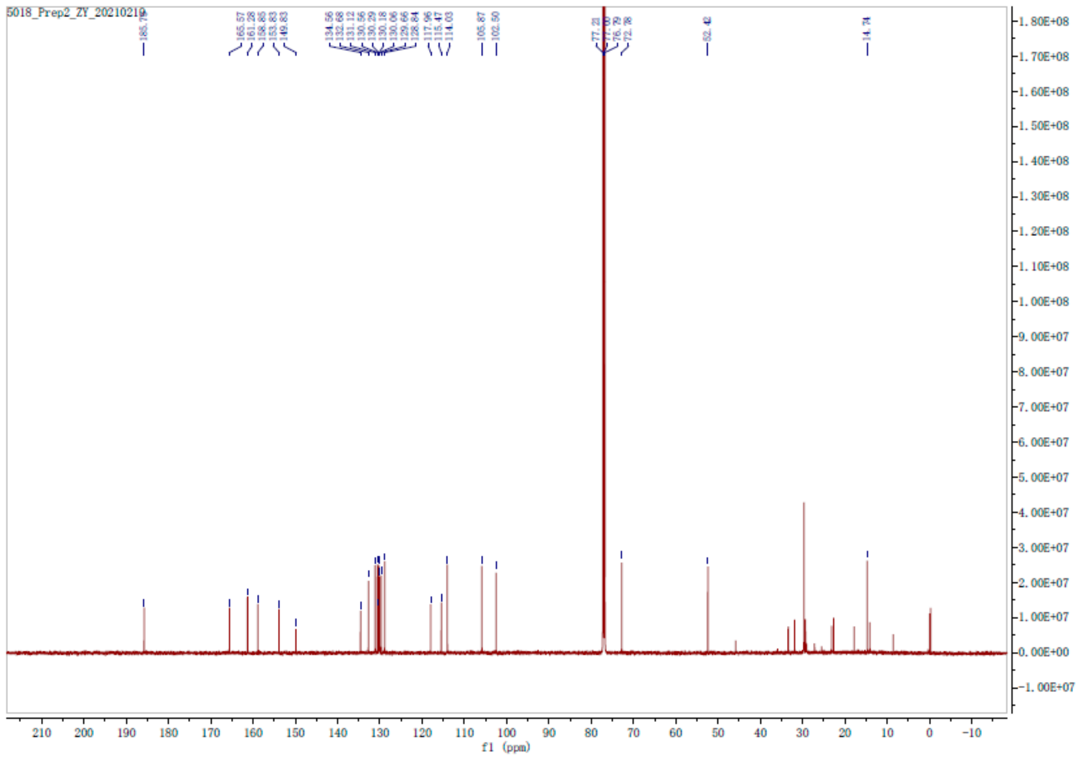

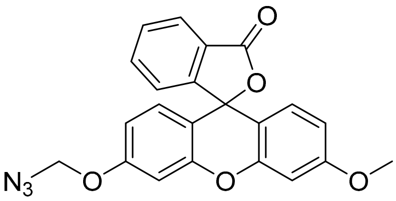

Compound D

^{1}H NMR (600 MHz, $CDCl_3$) δ 8.25 (dd, *J* = 7.9, 1.0 Hz, 1H), 7.74 (td, *J* = 7.5, 1.2 Hz, 1H), 7.68 (td, *J* = 7.8, 1.2 Hz, 1H), 7.31 (dd, *J* = 7.5, 0.8 Hz, 1H), 7.07 (d, *J* = 2.4 Hz, 1H), 6.91 (d, *J* = 8.9 Hz, 1H), 6.84 (d, *J* = 9.7 Hz, 1H), 6.80 (dd, *J* = 8.9, 2.4 Hz, 1H), 6.54 (dd, *J* = 9.7, 1.9 Hz, 1H), 6.45 (d, *J* = 1.9 Hz, 1H), 5.24 (s, 2H), 3.65 (s, 3H). ^{13}C NMR (151 MHz, $CDCl_3$) δ 185.82, 165.54, 160.33, 158.75, 153.73, 149.42, 134.48, 132.76, 131.18, 130.56, 130.29, 130.19, 129.74, 129.04, 118.36, 116.20, 113.55, 106.02, 102.84, 79.41, 52.44. HRMS (ESI) M/Z 403.1122 ([M + H^+]), calculated for [$C_{22}H_{15}N_3O_5$ + H^+] 402.1090.

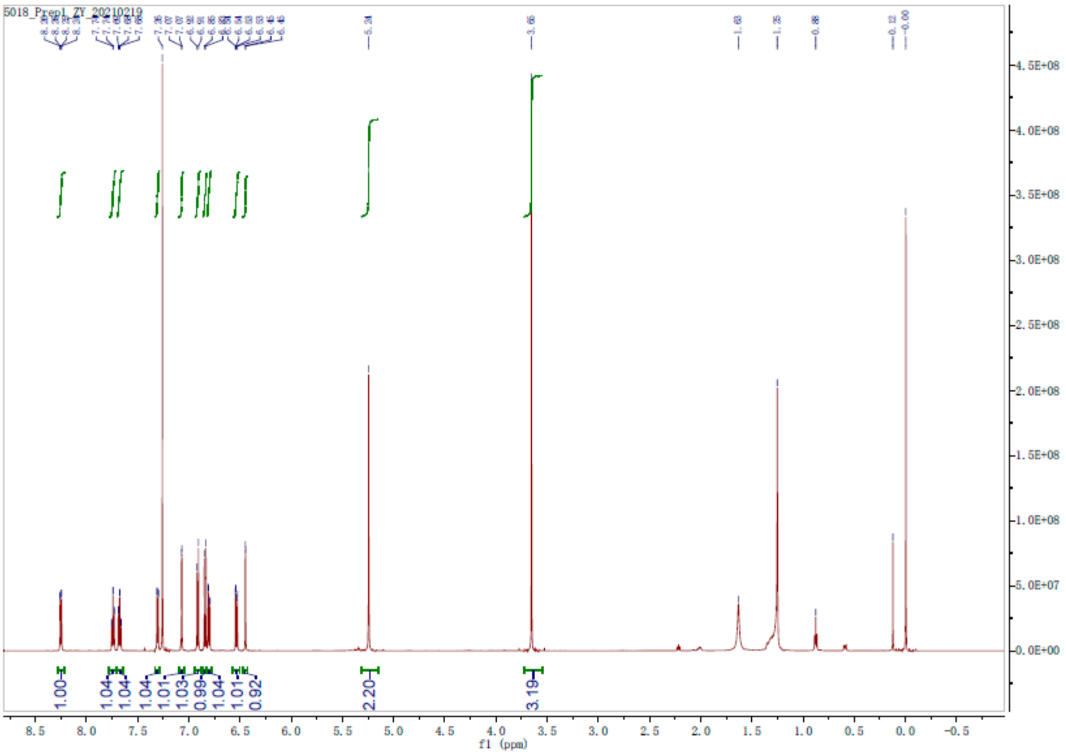
5018_Prep1_ZY_20210219
5.34
3.66
1.63
1.35
0.88
0.12
0.00
1.00
1.04
1.04
1.04
1.01
1.03
0.99
1.04
1.01
0.92
2.20
3.19
f1 (ppm)

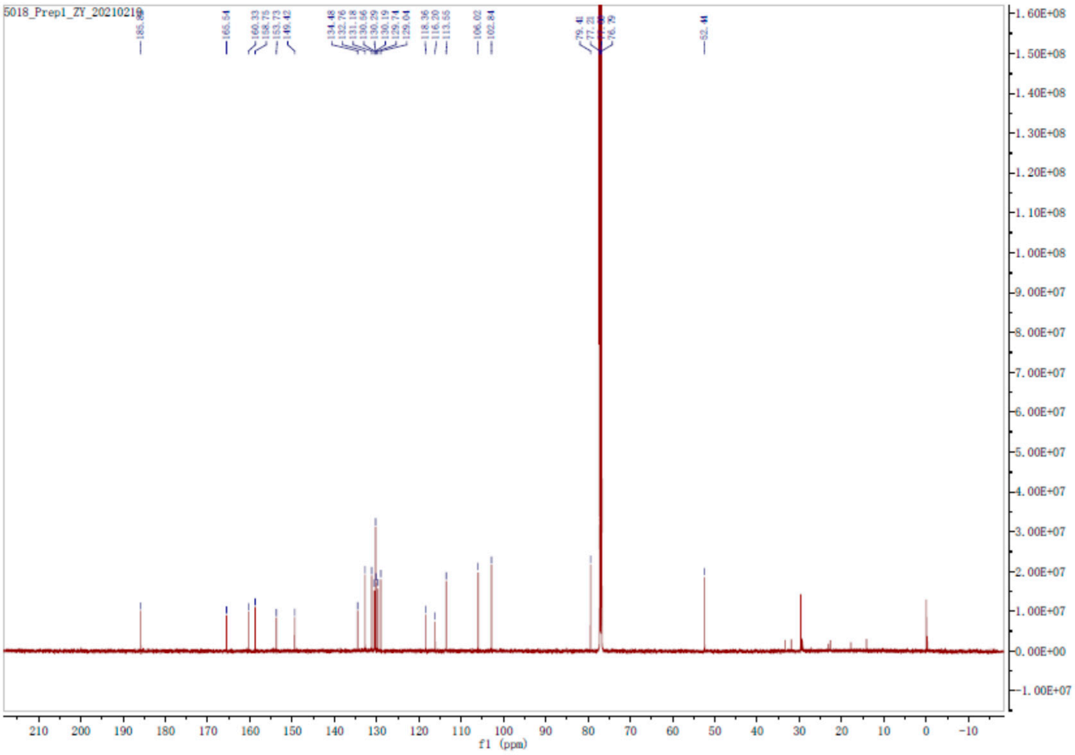
5018_Prep1_ZY_20210219
185.89
165.54
160.33
158.75
153.73
149.42
134.48
132.76
131.18
130.56
130.29
130.19
129.74
129.04
118.36
116.20
113.55
106.02
102.84
79.41
77.23
76.79
52.41
f1 (ppm)

Compound C 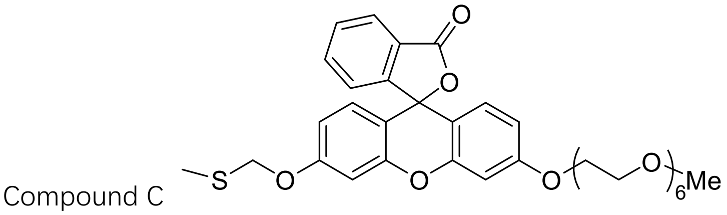

^{1}H NMR (600 MHz, CD_3OD_SPE) δ 8.01 (d, *J* = 7.7 Hz, 1H), 7.77 (td, *J* = 7.5, 1.1 Hz, 1H), 7.71 (td, *J* = 7.6, 0.8 Hz, 1H), 7.20 (d, *J* = 7.7 Hz, 1H), 6.91 (dd, *J* = 16.7, 2.4 Hz, 2H), 6.75 – 6.65 (m, 4H), 5.26 (s, 2H), 4.18 – 4.17 (m, 2H), 3.89 – 3.82 (m, 2H), 3.71 – 3.67 (m, 2H), 3.65 – 3.63 (m, 2H), 3.63 – 3.56 (m, 14H), 3.51 – 3.48 (m, 2H), 3.32 (s, 3H), 2.22 (s, 3H). ^{13}C NMR (151 MHz, CD_3OD_SPE) δ 171.30, 162.28, 160.43, 154.44, 153.79, 153.64, 136.71, 131.24, 130.10, 130.06, 127.92, 125.85, 125.23, 114.18, 113.31, 113.26, 112.50, 104.27, 102.75, 84.86, 73.60, 72.94, 71.80, 71.60, 71.54, 71.51, 71.33, 70.64, 69.09, 59.08, 14.47. HRMS (ESI) M/Z 671.2526 ([M + H^+]), calculated for [$C_{35}H_{42}O_{11}S + H^+$] 671.2448.

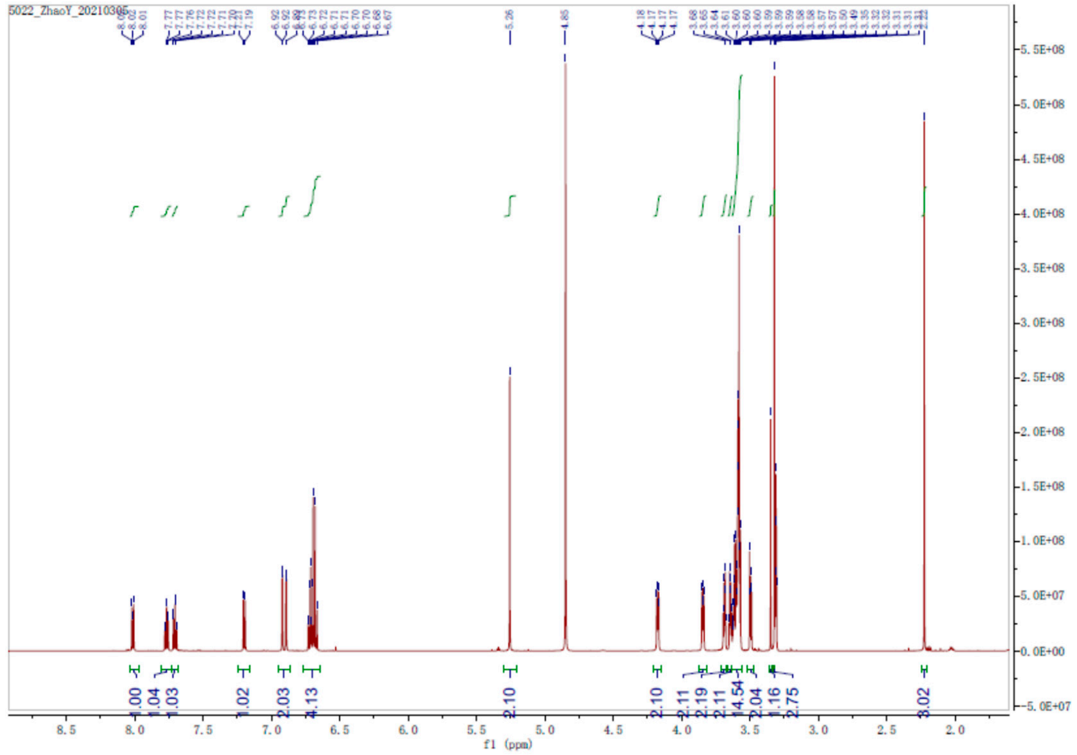

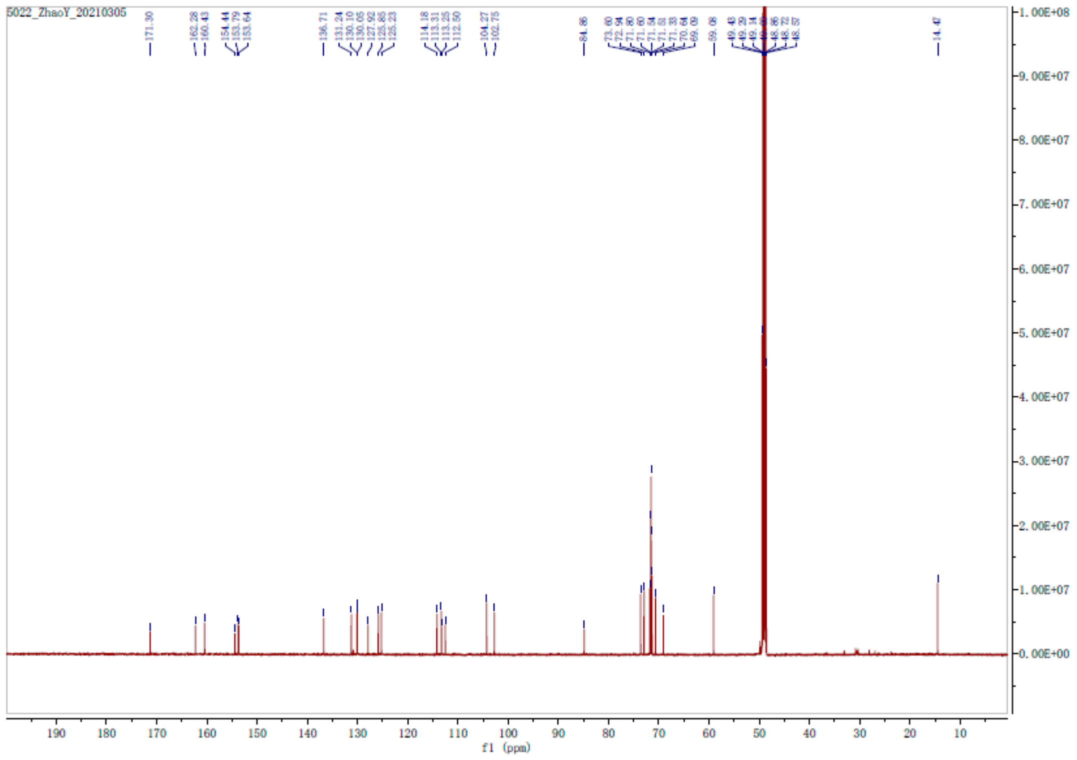

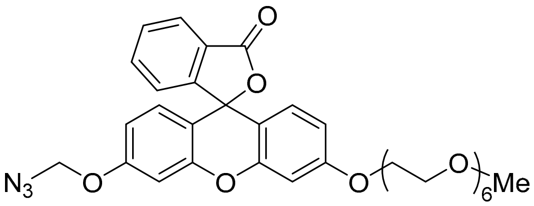

FL-N_3

^{1}H NMR (600 MHz, CD_3OD_SPE) δ 8.02 (d, *J* = 7.7 Hz, 1H), 7.77 (td, *J* = 7.5, 1.1 Hz, 1H), 7.71 (td, *J* = 7.6, 0.8 Hz, 1H), 7.20 (d, *J* = 7.7 Hz, 1H), 6.97 (d, *J* = 2.4 Hz, 1H), 6.90 (d, *J* = 2.4 Hz, 1H), 6.81 – 6.66 (m, 4H), 5.30 (s, 2H), 4.19 – 4.17 (m, 2H), 3.87 – 3.83 (m, 2H), 3.70 – 3.68 (m, 2H), 3.66 – 3.63 (m, 2H), 3.63 – 3.56 (m, 14H), 3.51 – 3.48 (m, 2H), 3.32 (s, 3H). ^{13}C NMR (151 MHz, CD_3OD_SPE) δ 181.51, 171.22, 162.30, 159.82, 154.39, 153.70, 136.75, 131.29, 130.42, 130.10, 127.84, 125.89, 125.20, 114.30, 113.75, 113.43, 112.43, 104.35, 102.75, 84.57, 80.67, 72.93, 71.79, 71.69, 71.63, 71.60, 71.32, 70.63, 69.10, 59.07. HRMS (ESI) M/Z 666.2670 ($[M + H^+]$), calculated for $[C_{34}H_{40}N_3O_{11} + H^+]$ 666.2585.

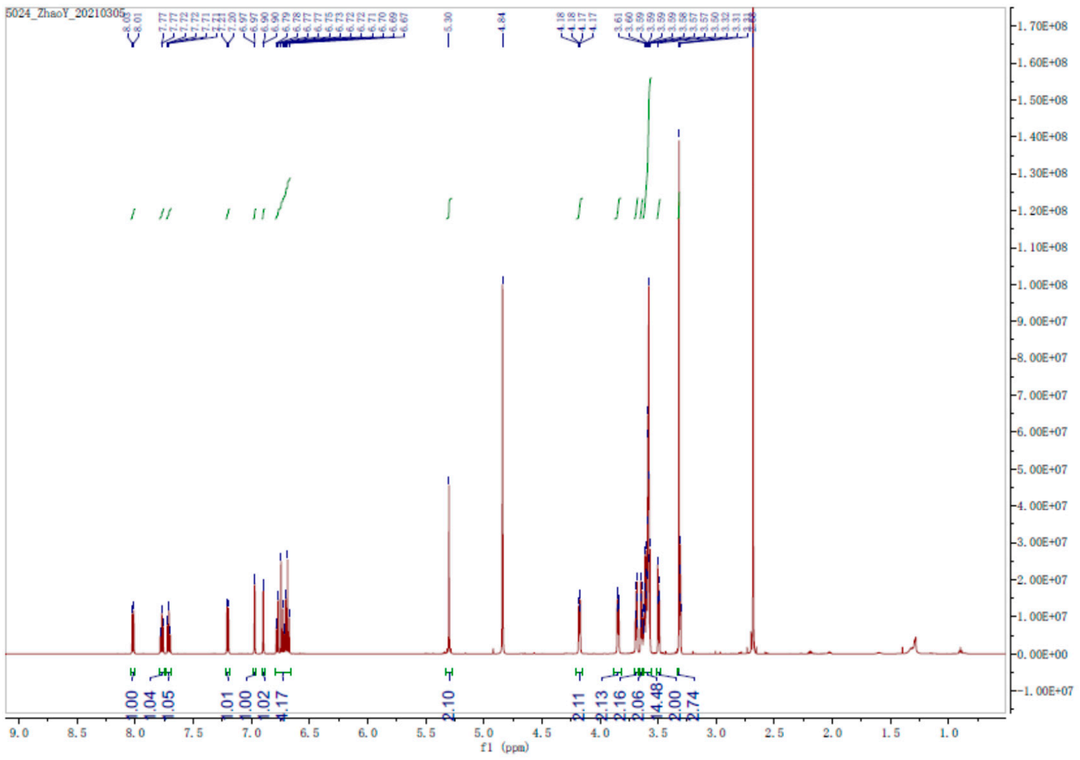
5024_ZhaoY_20210305
f1 (ppm)

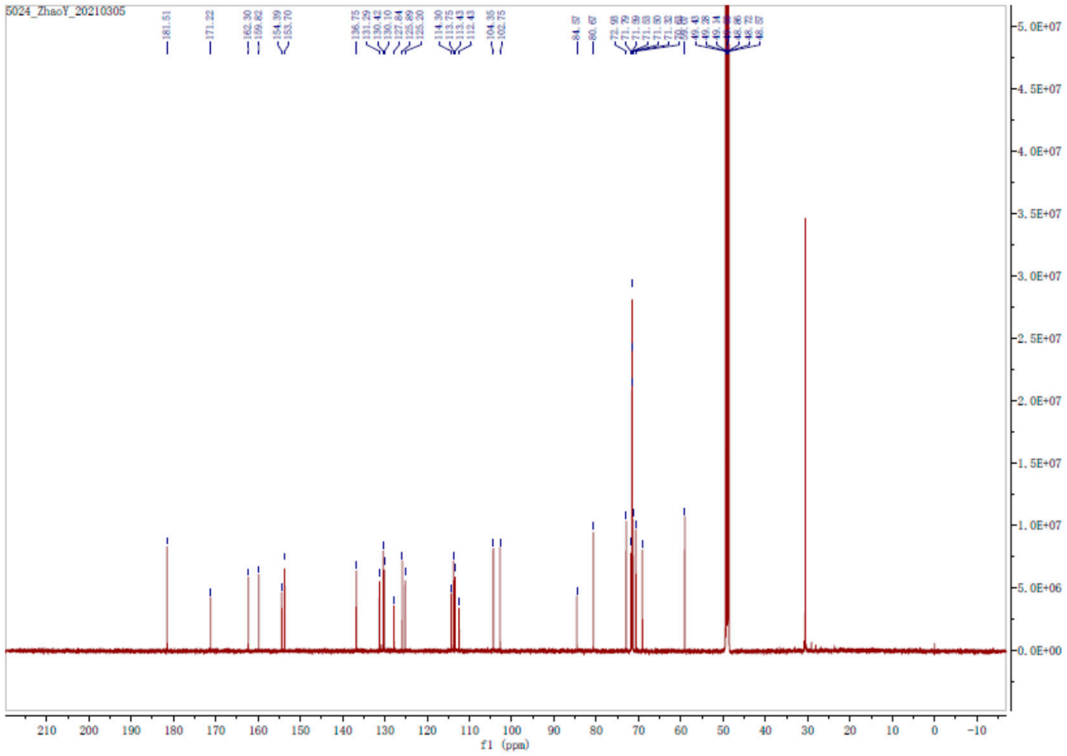
5024_ZhaoY_20210305
f1 (ppm)
